# Antimicrobial Efficacy of Ampicillin With Ceftriaxone in Comparison With Diclofenac Sodium, Modified Triple Antibiotic Paste, and Calcium Hydroxide Against Enterococcus faecalis: An In-Vitro Study

**DOI:** 10.7759/cureus.76276

**Published:** 2024-12-23

**Authors:** Ravali Mekala, Swathi Aravelli, Uday K Podugu, Sivaram Penigalapati, Rukmini A Masuna, Divyasree Vaddempudi

**Affiliations:** 1 Dentistry, Malla Reddy Dental College for Women, Hyderabad, IND; 2 Conservative Dentistry and Endodontics, Malla Reddy Dental College for Women, Malkajgiri, IND; 3 Conservative Dentistry and Endodontics, Malla Reddy Dental College for Women, Hyderabad, IND

**Keywords:** antimicrobial efficacy, calcium hydroxide paste, enterococcus faecalis, intracanal medicament, triple antibiotic paste

## Abstract

Objective

This in vitro study aimed to assess and compare the antimicrobial effectiveness of ampicillin with ceftriaxone (AC), diclofenac sodium (DS), modified triple antibiotic paste (MTAP), and calcium hydroxide (CH) against *Enterococcus faecalis* in root canal systems.

Materials and methods

The antimicrobial activity of the medicaments was assessed by determining the minimum inhibitory concentrations (MIC) via the agar well diffusion method. A total of 40 extracted permanent teeth underwent root canal treatment, and *Enterococcus faecalis* was introduced into the canal preparations. Intracanal medicaments, including AC, DS, MTAP, and CH, were applied, and their antimicrobial effects were quantified by colony-forming unit (CFU) counts before and after application. The impact on bacterial viability was further examined using confocal laser microscopy on root canal sections treated with different medicaments.

Results

The MIC results indicated lower concentrations of AC and MTAP, suggesting higher efficacy. AC demonstrated a more significant reduction in CFU counts than DS, MTAP, and CH. Confocal laser microscopy further supported the superior antimicrobial activity of AC.

Conclusion

The MIC results revealed that AC and MTAP required lower concentrations to inhibit *Enterococcus faecalis*, indicating higher antimicrobial efficacy. Among the medicaments tested, AC significantly reduced CFU counts more than DS, MTAP, and CH. Confocal laser microscopy analysis further confirmed the superior antimicrobial activity of AC by showing a greater reduction in bacterial viability in the treated root canal sections.

## Introduction

Periapical infections and root canal treatment failure are commonly linked to bacterial colonization and the persistence of microbial byproducts in the root canal system. The success of root canal therapy mostly depends on the effective reduction of microbial load to prevent reinfection and promote optimal healing. To achieve bacterial reduction, various strategies, including mechanical instrumentation, irrigation protocols, and intracanal medicaments, have been employed to ensure thorough disinfection and minimize residual bacteria in the root canal [1]. Achieving a bacteria-free environment prior to obturation is critical for the long-term success of endodontic treatment [2].

*Enterococcus faecalis* is frequently implicated in the failure of endodontic treatments [3-7], largely due to its capacity to form resilient biofilms that protect it from phagocytosis and make it highly resistant to conventional antimicrobial agents. Given the polymicrobial nature of endodontic infections, reliance on a single empirical antibiotic is often inadequate for eradicating the diverse array of pathogens present. Thus, using combination antibiotics is essential to effectively target a broad spectrum of endodontic pathogens and to reduce the risk of developing microbial resistance.

This study aimed to evaluate the antimicrobial efficacy of various intracanal medicaments against* Enterococcus faecalis*, with an emphasis on a novel approach involving a combination of ampicillin and ceftriaxone (AC). This dual therapy could provide an innovative strategy for addressing microbial resistance in endodontic treatments. The study's primary objectives were to determine the minimum inhibitory concentration (MIC), assess the antimicrobial synergy between the two agents (AC), compare the efficacy of different intracanal medicaments against *Enterococcus faecalis (E. faecalis)*, and examine bacterial viability using confocal laser scanning microscopy. By achieving these objectives, the study aims to offer critical insights into developing effective intracanal medicaments, enhancing the success of endodontic procedures, and improving patient outcomes.

Aim

This study aimed to evaluate and compare the antimicrobial efficacy of a novel intracanal medicament, a combination of ampicillin with ceftriaxone (AC), with traditional medicaments, such as diclofenac sodium (DS), calcium hydroxide (CH), and modified triple antibiotic paste (MTAP), specifically targeting *Enterococcus faecalis*.

## Materials and methods

**Figure 1:**
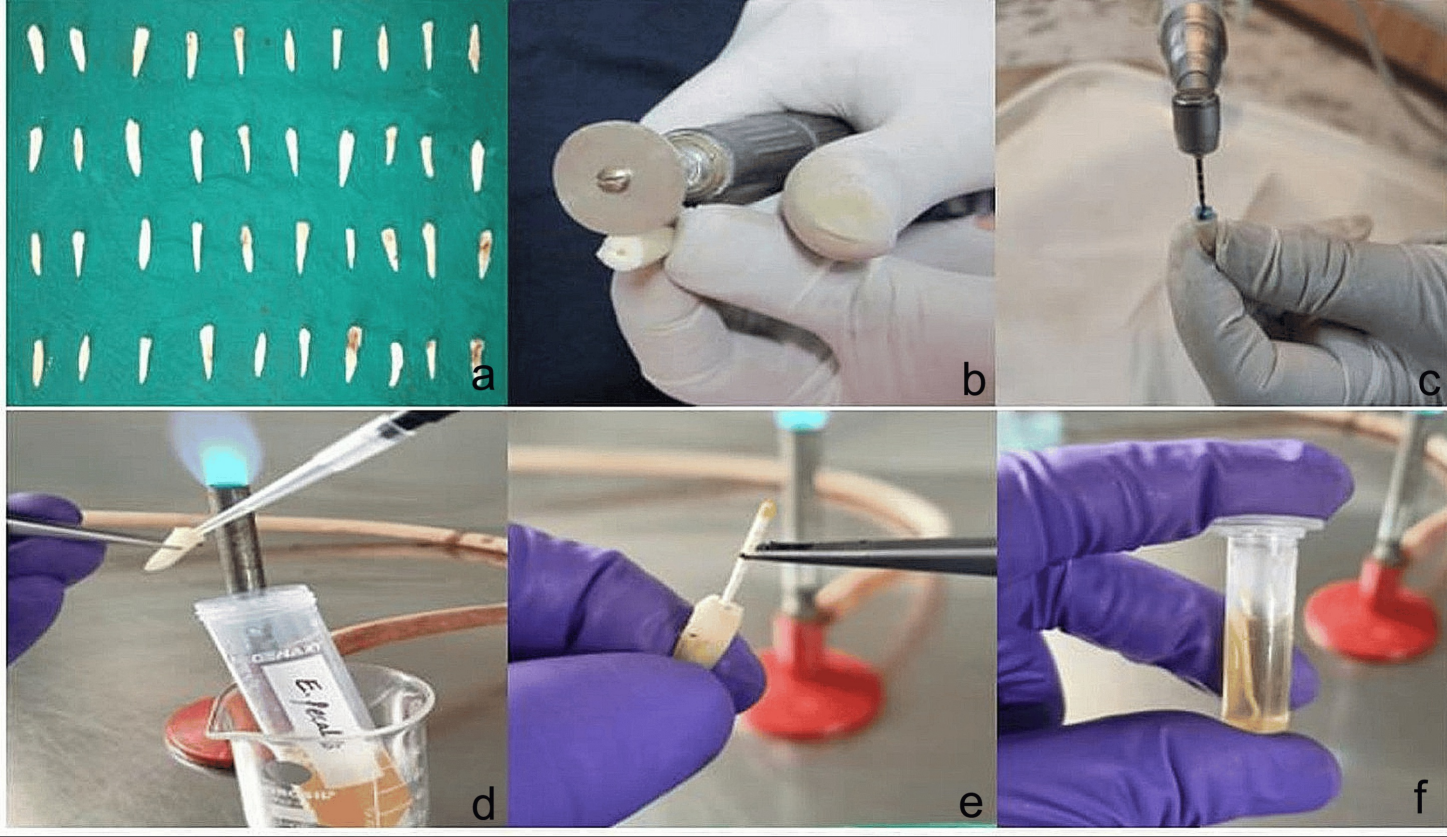
(a) 40 extracted single rooted permanent teeth were taken; (b) Decoronated with diamond disc; (c) The roots were subjected to standardized instrumentation using rotary up to F3-ProTaper; (d) After sterilization, root canals are inoculated with pure culture of E. faecalis; (e) Sterile absorbent paper point was inserted into the root canal and left for five minutes; (f) Paper points were aseptically transferred to Eppendorf tubes containing 1ml thioglycollate broth

A total of 40 extracted, single-rooted permanent teeth were selected for the study. The teeth were decoronated using a diamond disc to standardize the preparation. Later, the working length was determined by inserting a #10 K-file (Mani) into each root canal. The canals were then subjected to standardized instrumentation using rotary files up to F3-ProTaper (Dentsply-Maillefer). During biomechanical preparation, the root canals were irrigated with 2.5% sodium hypochlorite (NaOCl). Final irrigation was performed using 2 mL of 17% ethylenediaminetetraacetic acid (EDTA), which was allowed to remain in the canals for one minute to remove the smear layer, followed by a rinse with 2 mL of saline solution. The canals were then dried using size 30 absorbent paper points, and the apical foramen was sealed with composite resin. For sterilization, the prepared tooth samples were autoclaved at 121°C for 20 minutes at 20 psi. Two autoclaving cycles were performed, and bacterial viability was assessed to confirm the sterility of the samples (Figure 1) [8-11].

Inoculation of the pathogen into root canal preparations:

A pure isolated *Enterococcus faecalis* ATCC 29212 colony was selected from an agar plate and inoculated into an autoclaved sterile brain-heart infusion (BHI) broth. The inoculated culture was incubated at 37°C for 24 hours to obtain a fresh, active pathogen culture. Sterilized root canal samples were subsequently inoculated with this pure *E. faecalis* culture, and the samples were incubated for seven days. Specifically, 10 µL of the bacterial inoculum was introduced into each sterile root canal, and the samples were placed in sterile Eppendorf tubes. Each tube was filled with 1 mL of the active *E. faecalis* culture (1.5 x 108/mL, equivalent to a 0.5 McFarland standard), and the tubes were incubated at 37°C for seven days to allow for bacterial colonization and the infection of the root canal.

After seven days of incubation, the tooth samples were removed from the Eppendorf tubes, and 5 mL of sterile saline was used to rinse the incubation broth from each sample. A size 20 sterile absorbent paper point (DENTSPLY, India) was inserted into the root canal for five minutes to absorb residual bacteria. The paper points were subsequently transferred to test tubes containing 1 mL of Thioglycollate broth, from which serial dilutions were prepared (Figure 1). A 0.1 mL aliquot of each dilution was plated onto Mueller-Hinton agar plates and incubated for 24 hours. After incubation, the number of colony-forming units (CFUs) was counted. The samples were then randomly divided into four groups with n=10 in each group, based on the type of intracanal medicament applied [11,12]: Group 1 - ampicillin ceftriaxone (AC), group 2 - diclofenac sodium (DS), group 3 - modified triple antibiotic paste (MTAP), and group 4 - calcium hydroxide as the control group (CH).

Determination of minimum inhibitory concentration of medicaments against *E. faecalis*:

Before evaluating the antimicrobial activity of the medicaments, the MIC was determined using the agar well diffusion method. A single bacterial colony of pure *E. faecalis* was transferred to a 150 mL conical flask containing 50 mL of broth media and incubated at 37°C for 8-12 hours. An assay was conducted using the pour plate technique in which 1% of the active bacterial culture was mixed with autoclaved agar media just before the solidifying temperature and poured into Petri dishes. Once the agar solidified, wells were created using a sterile well borer, and 100 µL of each sample was added to the wells. The plates were then incubated at 37°C for 18-24 hours.

**Figure 2:**
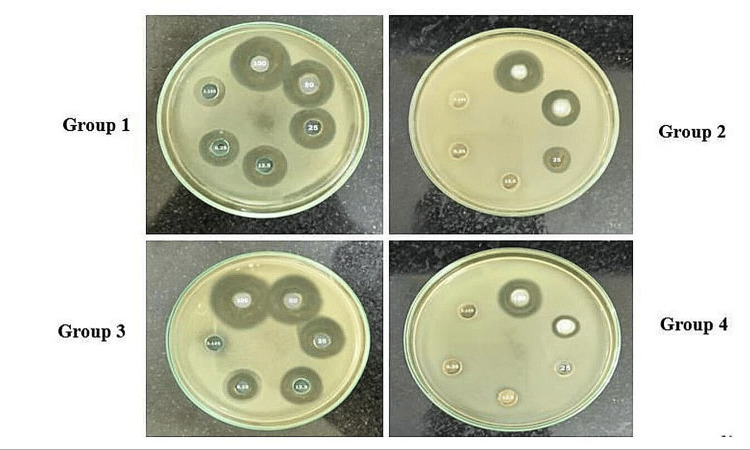
Determination of minimum inhibitory concentration Group 1 - minimum inhibitory concentration was 3.125 µg/mL; Group 2 - activity at 25 µg/mL; Group 3 - MIC was 6.25 µg/mL; Group 4 - the inhibitory activity was seen at 50mg/mL of sample concentration

After incubation, the plates were examined for inhibition zones, which were measured in millimeters. The results indicated that medicament groups 1 and 3 exhibited antimicrobial activity at lower concentrations. For group 1, a concentration of 3.125 µg/mL was sufficient to inhibit bacterial growth, while for group 3, 6.25 µg/mL was the minimum inhibitory concentration. In contrast, groups 2 and 4 showed less effectiveness, with group 2 demonstrating activity at 25 µg/mL, and group 4 (control) showing inhibition at 50 mg/mL. Based on these MIC values, the root canal infection model was established, and the antimicrobial activity of the medicaments was subsequently assessed (Table 1, Figure 2).

**Table 1:** Medicaments groups and concentration of drugs

Medicament groups	Concentration of medicaments					
	100	50	25	12.5	6.25	3.125
Ampicillin with ceftriaxone (AC)	22mm	18mm	16mm	14mm	12mm	10mm
Diclofenac sodium (DS)	18mm	12mm	10mm	-	-	-
Triple antibiotic paste (TAP)	20mm	18mm	16mm	14mm	12mm	-
Calcium Hydroxide (CH) (control group)	12mm	08mm	-	-	-	-

Application of medicaments in root canals

Intracanal medicaments were prepared on a sterile glass slab by mixing the test material with distilled water to achieve a paste-like consistency. The prepared medicaments were then introduced into the canal preparations using a lentulo spiral and sealed with a temporary restoration (Cavit, 3M ESPE, Germany). The samples were subsequently incubated at 37°C for seven days.

Assessment of antimicrobial efficacy of medicaments against E. *faecalis*

**Figure 3:**
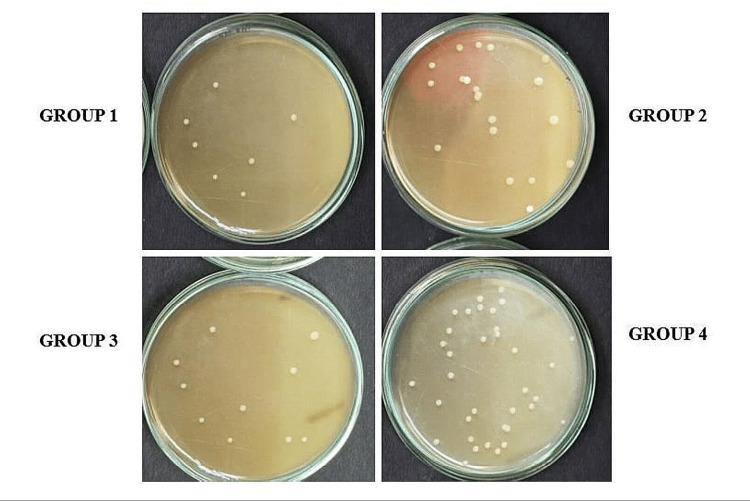
Microbiological analysis after treatment with test medicaments The number of colony-forming units was counted in each group after placing intracanal medicaments for seven days. Group 1 and group 3 showed fewer number of colonies of *E. faecalis* followed by group 2 and group 4.

After seven days, each tooth was irrigated with 5 mL of saline to remove the intracanal medicaments, and antimicrobial activity was evaluated by determining bacterial counts through paper point sampling. The efficacy of the medicaments was assessed by comparing the number of CFUs before (CFU-1) and after the placement of intracanal medicaments (CFU-2). *E. faecalis* was harvested from the root canals using sterile paper points. Each paper point was inserted into the canal for approximately five minutes and then aseptically transferred to an Eppendorf tube containing 1 mL of media. Serial dilutions were performed, and 0.1 mL of the diluted broth was plated onto agar plates, which were incubated at 37°C for 24-48 hours to allow for colony formation. The colonies were counted, and the results were compared to evaluate the antimicrobial efficacy of the different medicaments (Figure 3).

Observation of root canal sections by confocal laser scanning microscopy (CLSM)

**Figure 4:**
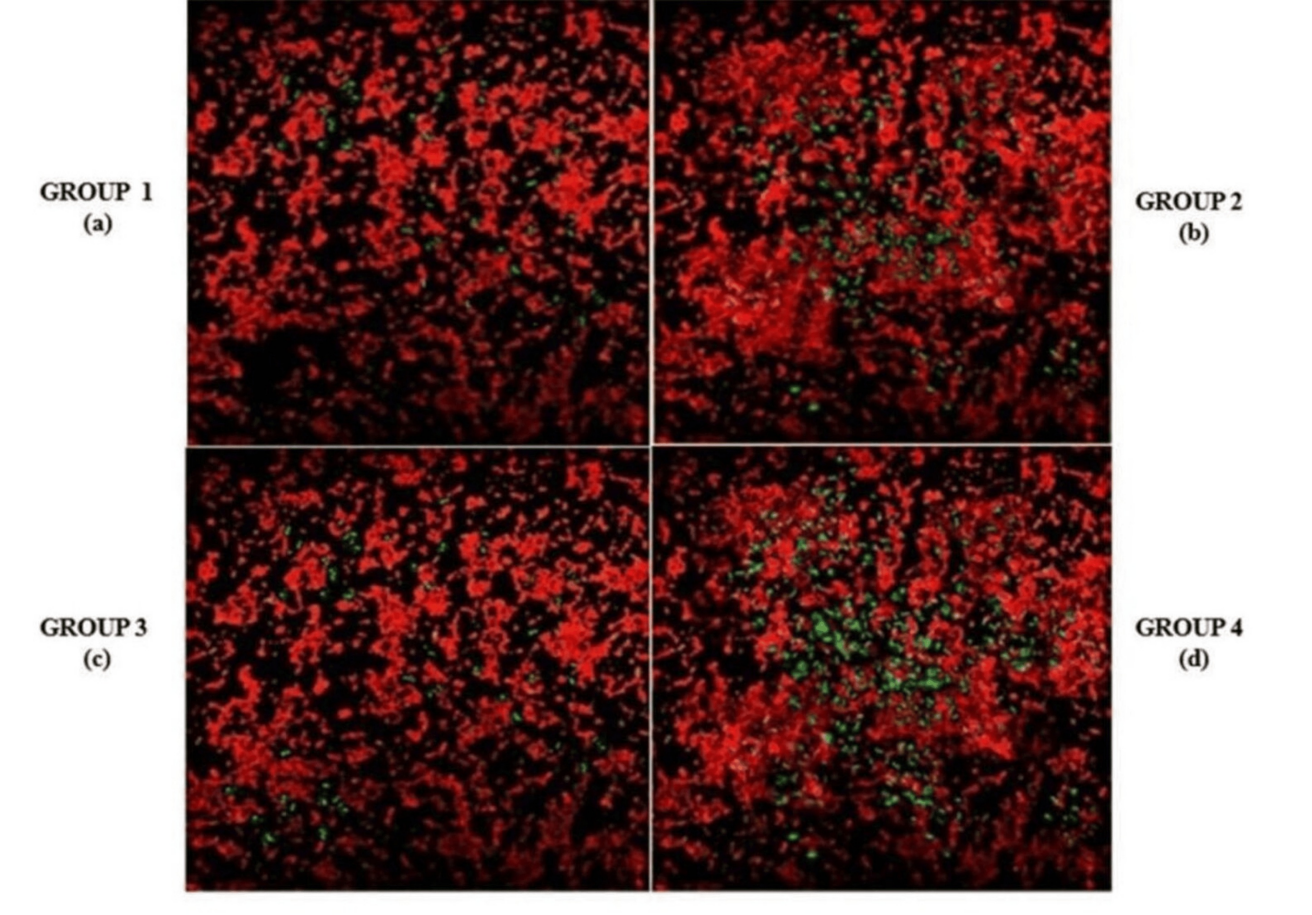
Confocal laser scan microscopy images showing live and dead bacteria (green fluorescence - live bacteria, red fluorescence - dead bacteria) (a) Ampicillin + ceftriaxone; (b) Diclofenac sodium; (c) Modified triple antibiotic paste; (d) Calcium hydroxide

On day seven, the root canal samples were examined under CLSM to assess the viability of bacteria, with live bacteria appearing green and dead bacteria red. The teeth were longitudinally sectioned into 1 mm thick slices, which were then stained with fluorescent dyes, SYTO 9 (for live bacteria), and propidium iodide (for dead bacteria) (Baclight, Carsland, CA, USA). The stained sections were observed under CLSM to evaluate the bacterial viability in the root canal. The results showed a higher percentage of dead bacteria in group 1 and group 3, followed by group 2 and group 4, respectively (Figure 4).

Statistical analysis

Statistical analysis was performed using SPSS software version 20 (IBM Inc., Armonk, NY). Descriptive statistics, including mean and standard deviation, were calculated for all data. The Kruskal-Wallis ANOVA test was used to compare the antimicrobial efficacy among the four groups. The Mann-Whitney U-test was employed for pairwise comparisons, while the Wilcoxon matched-pairs test was used for within-group comparisons.

**Table 2:** Comparison between colony forming units counts at baseline (CFU1) and after seven days (CFU2) in the groups, along with percentage change, and inter-group comparison CFU - colony-forming unit

Groups	CFU counts	Means (SD)	% change	P		Pair-wise comparison (Mann-Whitney U-test)	
Group 1	CFU1	150.66 (±5.13)	95.58	0.0018	1 vs 2	P = 0.2532	No statistically significant difference
	CFU2	06.66 (±1.5)			1 vs 3	P = 9671	No statistically significant difference
Group 2	CFU1	146.00 (±5.29)	92.24	0.0083	2 vs 3	P = 0.0081	Very strong evidence (p ˂ 0.001) against null hypothesis
	CFU2	11.33 (±3.51)			2 vs 4	P = 0.3122	No statistically significant difference
Group 3	CFU1	153.33 (±3.51)	94.14	0.0041	3 vs 4	P = 0.0510	Very strong evidence (p ˂ 0.001 to 0.01) against null hypothesis
	CFU2	09.00 (±2.64)					
Group 4	CFU1	150.66 (±4.5)	83.41	0.1954	1 vs 4	P = 0.0113	Very strong evidence (p ˂ 0.001 to 0.01) against null hypothesis
	CFU2	25.00 (±1.73)					

**Figure 5:**
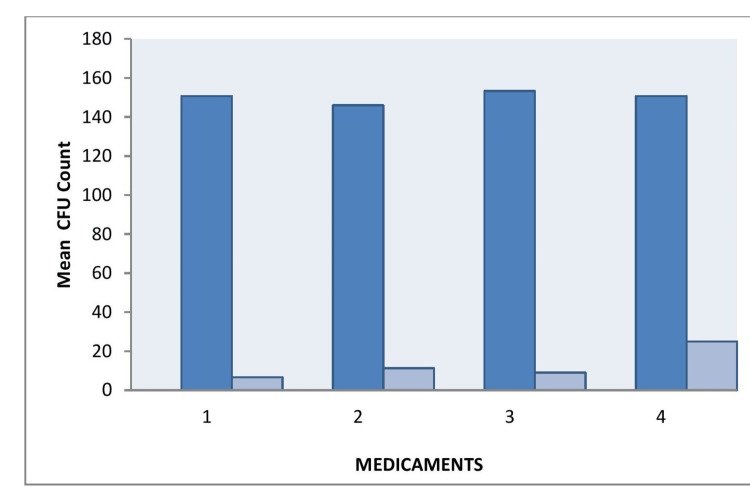
Comparison between colony-forming unit-1 and colony-forming unit-2 CFU - colony-foming unit

**Figure 6:**
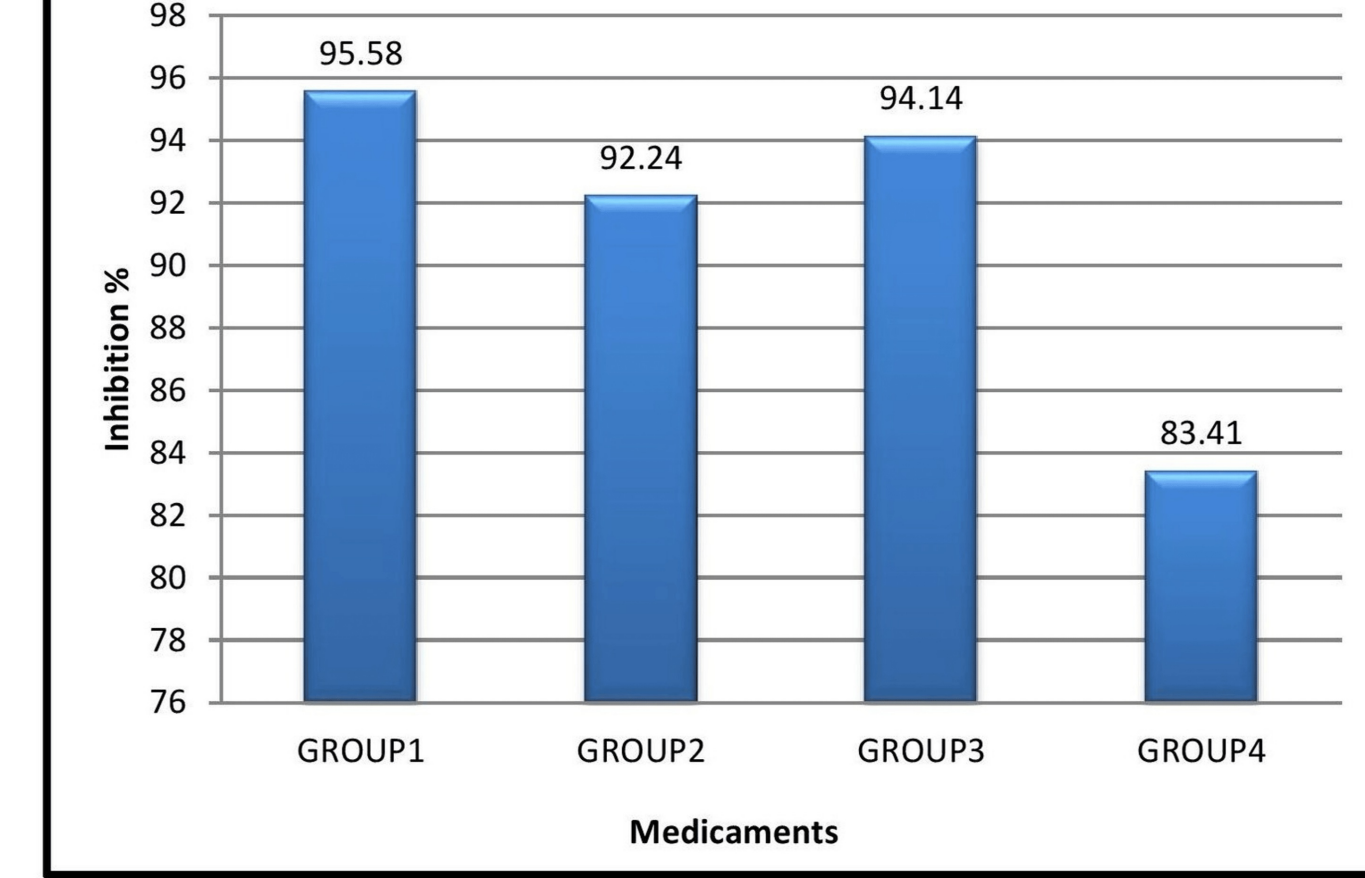
Efficacy of medicaments represented as percentage inhibition of E. faecalis

## Results

The results of this study indicated significant differences in the antimicrobial efficacy of the intracanal medicaments evaluated against *Enterococcus faecalis*. The MIC values for the different medicaments were as follows: group 1 (AC) had the lowest MIC of 3.125 µg/mL, followed by group 3 (MTAP) at 6.25 µg/mL, group 2 (DS) at 25 µg/mL, and group 4 (CH) at 50 mg/mL. Significant reductions in CFUs were observed in group 1, especially at lower concentrations, with group 3 showing the next most substantial decrease in bacterial load. Confocal laser microscopy analysis further confirmed the superior antimicrobial activity of AC by showing a greater reduction in bacterial viability in treated root canal sections.

Statistical analysis revealed that p-values <0.05 were considered significant, confirming the differences in antimicrobial effectiveness. The data showed that group 1 (AC) exhibited the most significant antimicrobial activity against *Enterococcus faecalis* at lower concentrations, followed by group 3 (MTAP), group 2 (DS), and group 4 (CH) (Figures 5, 6; Table 2).

## Discussion

The effective control of endodontic pathogens and their byproducts is critical for the success of root canal therapy. A complete eradication of these pathogens is essential to prevent severe tooth damage and avoid a relapse of infection. Despite thoroughly cleaning and shaping the root canal system, some microbes may evade treatment and cause reinfection. Reinfection is often due to pathogens residing in the deeper, inaccessible regions of the root canals where irrigants and medicaments cannot reach, thereby allowing these bacteria to survive and propagate.

*Enterococcus faecalis*, a gram-positive facultative anaerobe, is one of the most common pathogens responsible for endodontic treatment failure. This microorganism is notorious for its ability to form resistant biofilms, which protect it from conventional endodontic procedures. *E. faecalis* can persist in low-nutrient environments and tolerate extreme conditions, such as high alkaline pH (up to 11.5), which is often encountered in the root canal during treatment. Its small size allows it to penetrate deep into dentinal tubules, where it can survive as a solitary organism, often without the need for coexisting bacteria [4]. Furthermore, *E. faecalis* exhibits remarkable resistance to bile salts, detergents, heavy metals, ethanol, azide, and desiccation, contributing to its persistence in the root canal system. It can grow at temperatures ranging from 10°C to 45°C and can endure exposure to 60°C for up to 30 minutes.

CH, commonly used in endodontics for its antimicrobial properties, works primarily by creating a highly alkaline environment that inactivates bacterial membrane enzymes. However, *E. faecalis* is notably resistant to CH, as its survival requires a pH of 11.5 or higher, which is difficult to achieve with CH alone. The pH of CH can only reach a maximum of 10.3 due to the buffering effect of dentin, and this pH gradient decreases further as it penetrates deeper into the dentinal tubules. Accordingly, *E. faecalis* remains viable in the deeper portions of the canal, where the pH remains relatively stable due to dentin's buffering action, thereby limiting the effectiveness of CH in eliminating this pathogen.

Modified triple antibiotic paste (MTAP), composed of a combination of clindamycin (500 mg), metronidazole (400 mg), and ciprofloxacin (500 mg), has emerged as an effective intracanal medicament (ICM) for eliminating bacteria from deeper areas of root canals. In this study, MTAP demonstrated superior efficacy to CH in reducing colony-forming units (CFUs). The antimicrobial activity of MTAP is attributed to the synergistic effects of its components, which target bacterial cell wall synthesis and DNA integrity. Studies have shown that nonsteroidal anti-inflammatory drugs (NSAIDs), particularly DS, also possess antibacterial properties, primarily by inhibiting bacterial DNA synthesis and disrupting membrane function. Diclofenac and ibuprofen have exhibited antibacterial activity against *E. faecalis*, with diclofenac showing significant potential [9,10].

In this study, the combination of AC demonstrated the most potent antimicrobial activity against *E. faecalis*, followed by MTAP, DS, and CH. The MIC values for group 1 (AC) and group 3 (MTAP) were notably low, indicating strong antimicrobial efficacy at lower concentrations. Furthermore, a zone of inhibition exceeding 20 mm was observed with the AC combination, highlighting the effective activity of these antibiotics at minimal concentrations. The high efficacy of AC can be attributed to the broad-spectrum antimicrobial properties of ampicillin, a medication that targets both Gram-positive and Gram-negative bacteria. A type of aminopenicillin, ampicillin interferes with bacterial cell wall synthesis by binding to penicillin-binding proteins (PBPs), thus inhibiting peptidoglycan synthesis.

A third-generation cephalosporin, ceftriaxone similarly disrupts cell wall synthesis and exhibits bactericidal activity. When used together, ampicillin and ceftriaxone form a double β-lactam regimen increasingly recognized as an effective alternative to ampicillin and gentamicin combinations in treating *E. faecalis* infective endocarditis, as recommended by international guidelines [11]. The synergy between AC likely results from complementary binding to different PBPs: ampicillin binds to PBPs 4 and 5, while ceftriaxone binds to PBPs 2 and 3 [12]. This combined action leads to a complete saturation of the bacterial cell wall synthesis, enhancing the antimicrobial efficacy of the treatment.

This study's findings indicate that antibiotic combinations, specifically AC and MTAP, exhibit significantly enhanced efficacy against *E. faecalis* compared to single-agent medicaments, such as CH and DS. This is the first study to evaluate the use of AC as an intracanal medicament for *E. faecalis*, and the results show promising potential for these antibiotics in endodontic therapy. However, further research is necessary to evaluate the duration of action of this combination of drugs and its effectiveness against a broader spectrum of endodontic pathogens. Future studies should also investigate the potential for resistance development and the impact of these medicaments on surrounding tissues in in-vivo studies.

## Conclusions

The results of this study, based on MIC assessments, CFU quantification (CFU1 and CFU2), and confocal laser scanning microscopy, clearly demonstrate that group 1 (AC) exhibited significant antimicrobial activity against *E. faecalis* at lower concentrations. This was followed by group 3 (MTAP), group 2 (DS), and group 4 (CH) in terms of efficacy. These findings highlight the potential of AC as an effective intracanal medicament in endodontic treatments. Nevertheless, further research is needed to explore the duration of action of this combination, its effects on other bacterial species, and its potential clinical applicability in endodontic practice.
